# Foot-and-Mouth Disease Vaccines by Design; Production of Capsid-Modified Foot-and-Mouth Disease Viruses with Improved Cell Culture Growth

**DOI:** 10.3390/vaccines13030281

**Published:** 2025-03-06

**Authors:** Stephen Berryman, Femke Feenstra, Amin Asfor, Jose Coco-Martin, Terry Jackson, Tobias J. Tuthill

**Affiliations:** 1 Pirbright Institute, Ash Road, Woking GU24 0NF, UK; a.asfor@surrey.ac.uk (A.A.); tjackson57@myyahoo.com (T.J.); 2 Boehringer Ingelheim Animal Health Netherlands BV, P.O. Box 65, 8200 AB Lelystad, The Netherlands; feenstra_f@hotmail.com (F.F.); jose.coco-martin@boehringer-ingelheim.com (J.C.-M.)

**Keywords:** FMDV, vaccine, tissue culture adaptation, reverse genetics, heparan sulfate

## Abstract

Background/Objectives: Vaccination is important for controlling foot-and-mouth disease (FMD) in endemic regions and to lessen the effects of outbreaks in FMD-free countries. The adaptation of FMD virus to BHK cells is a necessary but time-consuming and costly step in vaccine production and can prove problematic for some isolates. Adaptation is, in part, driven by receptor availability and selects variants with altered receptor specificity that result from amino acid substitutions in the capsid proteins. Methods: To bypass the need for cell culture adaptation, we generated chimeric viruses with field-strain capsids and introduced amino acid substitutions associated with cell culture adaptation. We targeted two sites on the capsid: the canonical heparan sulphate binding site and the icosahedral 5-fold symmetry axes. Results: Our results show that some viruses with unmodified wild-type (wt) capsids grew well in BHK cells (suspension and adherent), whereas others showed poor growth. For viruses that showed good growth, the introduction of amino acid changes associated with cell culture adaptation improved the rate of growth but not virus titres or yields of 146S particles, whereas growth and 146S yields for viruses that grew poorly in BHK cells were greatly enhanced by some of the amino acid changes. For the latter viruses, the introduced changes did not appear to adversely affect virion stability or antigenicity. Conclusions: For FMD viruses that grow poorly in BHK cells, this approach could be a viable alternative to protracted adaptation by serial passage and could expedite the production of a new vaccine strain from a field virus.

## 1. Introduction

Foot-and-mouth disease (FMD) is highly contagious and caused by the FMD virus (FMDV), the type species of the *Aphthovirus* genus of the *Picornaviridae*, a family of small, single-stranded positive-sense RNA viruses. The FMDV genome is encased in an icosahedral capsid formed by 60 copies each of four structural proteins (VP1–VP4). The outer capsid is formed by VP1–VP3; VP1 surrounds the icosahedral 5-fold symmetry axes, while VP2 and VP3 alternate around the 3-fold symmetry axes [1,2]. To initiate an infection, the capsid proteins interact with cellular receptors. In vivo, FMDV field strains preferentially target epithelial cells, and use the epithelial cell-restricted integrin αvβ6 as the principal receptor [3,4,5,6,7]. FMDV binds αvβ6 via a conserved arginine-glycine-aspartic acid (RGD) motif in the GH-loop of VP1 and can also use other RGD-dependent integrins (αvβ1, αvβ3, and αvβ8) to initiate infection [8,9,10], but the role of these integrins in vivo is unclear.

FMD is endemic in many regions of the world and affects several wildlife species and also domesticated livestock, such as cattle, sheep, goats, and pigs [11,12]. FMD is associated with significant economic losses as a result of reduced productivity and trade restrictions imposed on affected countries [13]; thus, the impacts are likely to be greater for countries where FMD is endemic. For disease-free countries, the costs of maintaining a disease-free status can also be high, and they can also incur enormous financial losses as the consequence of a sporadic incursion [14]. Vaccination is important for controlling FMD in endemic regions and for lessening the effects of an outbreak in FMD-free countries [15]. FMDV exists as seven serotypes (O, A, C, Asia-1, and the Southern African Territories [SAT] serotypes, SAT-1, SAT-2, and SAT-3). This complicates efforts to control FMD via vaccination, as different vaccines are required for each serotype. Furthermore, within each serotype a large number of constantly evolving antigenic variants exist, which can lead to the emergence of novel strains [16,17]. This further complicates FMD control, as animals vaccinated with a specific strain may not be protected from infection by another virus of the same serotype, and new emerging strains may evade the immunity induced by the current vaccines [18]. Consequently, vaccines need to be a close antigenic match for the outbreak virus, which can necessitate the need to rapidly develop new vaccine strains to protect against emerging variants for which the existing vaccines are a poor match.

The current FMDV vaccines are chemically inactivated virus preparations produced in mammalian cell cultures in large industrial-scale fermenters. Baby hamster kidney cells (BHK-21 clone 13), that grow in suspension [19], are commonly used for vaccine production. However, BHK cells lack the αvβ6 integrin and can produce low yields of vaccine virus [7]. Thus, FMDV field strains may need to be adapted by serial passage to grow efficiently in BHK cell systems. The adaptation process is driven in part by the availability of cell surface receptors, and often selects for variants with amino acid (aa) changes on the outer surfaces of the capsids that allow the use of alternative, non-integrin receptors, such as heparan sulphate (HS), for cell attachment and entry [20,21,22,23,24,25,26,27,28,29].

Knowledge of the changes in the viral capsid that permit the use of non-integrin receptors could allow for the rational design of vaccine viruses that do not need to be adapted to BHK cells. Adaptation to HS receptors was first demonstrated for FMDV O1BFS [24], and subsequent studies identified the residue at VP3-56 as important for HS binding [27,30]. The structure of O1BFS bound to heparin (a structural analogue of HS) [30] showed that the HS binding site (here called the canonical HS binding site) is formed by a shallow depression in the outer capsid surface [30]. In the complex, heparin makes multiple contacts with all three outer capsid proteins, with the most prominent contacts made with R residues at VP3-56 and VP2-135 and a histidine (H) at VP1-195 [30]. In the FMDV field strain O1Kcar^2^, which does not bind HS, VP3-56 is H, while the other main heparin contact residues are conserved between O1BFS and O1Kcar^2^. Thus, for type O FMDV, the switch to HS binding appears to arise from only a small number of residue changes (including at VP3-56) that result in a net gain in positive charge in the depression that forms the HS binding site [27,30]. The structure of another cell-culture-adapted strain of FMDV, A1061, also in a complex with heparin, revealed a HS binding site in the same location on the capsid that is structurally very similar to that of O1BFS [22]. This suggests a common mechanism for cell culture adaptation; however, although the depression that forms the HS binding site is a conserved feature of FMDV capsids [2,22,31], other type A viruses, and viruses representative of other FMDV serotypes, do not appear to acquire the ability to bind HS at this site. Instead, cell culture adaptation selects for aa changes at other locations, in particular at a “hotspot” at the icosahedral, 5-fold symmetry axes of the capsid [25,26,28,32,33,34,35]. The aa substitutions at the 5-fold symmetry axes that are associated with altered receptor usage and improved virus growth in cell cultures often occur in the βF-βG (residues 83–85) and βD-βE (residues 108–112) loops of VP1 and involve positively charged aa substitutions. As five copies of VP1 surround the 5-fold symmetry axes, this creates a dense patch of positive charge on the outer capsid surface, which is thought to facilitate interactions with cell surface receptors. For example, a single glutamine (Q) to lysine (K) change at VP1-109 acquired during BHK cell passage enabled integrin-independent infection of CHO cells (which lack expression of the integrins used as receptors by FMDV) and improved virus growth in cell cultures by a virus with the capsid proteins from the A/Turkey/2/2006 field isolate [32]. Importantly, this same aa change also allowed for integrin-independent infection of CHO cells and improved virus growth in cell culture when it was introduced into FMDVs that had the capsids of type O (OUKG/35/2001) or Asia-1 (Asia-1/Bar/9/2009) field viruses [32]. Similarly for the SAT viruses, the acquisition of positively charged aa at VP1 110-112 and VP1 83-85 is associated with altered receptor usage and improved virus growth in cell cultures [25,26].

Adaptation of FMDV field strains to BHK cells can be time consuming, costly, and prove difficult for some viruses, and thus complicates efforts to develop a new vaccine to novel emerging strains. Here, we attempt to bypass the need for cell culture adaptation by generating chimeric FMDVs with capsids derived from field viruses (representative of type O, A, Asia-1, and SAT2) that include aa changes that occur during cell culture passage and characterise their growth in adherent and suspension BHK cells. The purpose of this approach is to improve virus growth under vaccine manufacturing conditions by the introduction of targeted amino acid changes to the capsid, rather than by protracted serial passage.

Our results show that the introduced changes increased the rate of virus growth in both adherent and suspension BHK cells. Importantly, we also show that for viruses that grow poorly in BHK cells, some aa changes can greatly improve yields of 146S (Svedberg units) intact virus particles under vaccine manufacturing conditions. In addition, we show that the stability and antigenicity of selected viruses was not adversely affected by the introduced changes. This approach has the potential to greatly expedite the production of new vaccine strains by eliminating the need for time-consuming and expensive cell culture adaptation. It may also reduce the need for extraneous agent testing, since viruses are produced in vitro rather than being isolated from field samples.

## 2. Materials and Methods

### 2.1. Cell Culture

Adherent baby hamster kidney (BHK-21 clone 13) cells (Pirbright Institute, Woking, UK) and IBRS-2 cells (Pirbright Institute, Woking, UK) were cultured in Glasgow’s modified Eagle’s medium (GMEM) (Sigma, Gillingham, UK) and Chinese hamster ovary (CHO) cells (ATCC CCL-61) in Ham’s F-12 (Sigma, Gillingham, UK), each supplemented with 10% foetal calf serum (FCS), 20 mM glutamine, penicillin (100 SI units/mL), and streptomycin (100 μg/mL). Primary bovine thyroid (BTY) cells and primary pig kidney cells were prepared and cultivated as described previously [36,37]. Boehringer–Ingelheim suspension baby hamster kidney (BHK-21) cells were cultured in a 5 L UniVessel^®^ Glass Bioreactor controlled by a BIOSTAT B controller (Sartorius Stedim Biotech, Aubagne, France), with a working volume of 2–4 L in BI Gibco Glasgow medium (GMEM) supplemented with 5% adult bovine serum (ABS). Cells were maintained under pH and dO2 control (pH 7.3, 40% dO2, 37 °C) and stirred continuously (450–800 rpm).

### 2.2. Infectious Copy Plasmids and Rescue of Recombinant Viruses

All of the infectious copy plasmids used in this study were based on pT7S3 [38], which contains a cDNA copy of the full-length viral RNA (vRNA) of FMDV O1Kaufbeuren (O1K). Chimeric viruses with wt capsids were generated by replacing the coding regions for VP2, VP3, VP1 and 2A of O1K with the corresponding region of a field virus. The construction of the infectious copy plasmids for viruses with wt capsids of A-Turkey/2/2006, Asia-1/Bar/9/2009 or OUKG/35/2001 (here called O1K-A WT, O1K-Asia WT and O1K-O WT, respectively) has been described previously [32]. Plasmids for the O1K-A KK, O1K-A RK, O1K-Asia KK, O1K-O KK and O1K-O RK viruses were generated by QuikChange Lightning site-directed mutagenesis (Agilent Technologies, Didcot, UK) using the appropriate infectious copy plasmid as the template and primer pairs, as described previously [32]. The infectious copy plasmids for the O1K-A HS, O1K-Asia RK, O1K-Asia HS, O_1_K-O HS, O1K-SAT2 WT, O1K-SAT2 KGR, O1K-SAT2 KKR, O1K-SAT2 KHR and O1K-SAT2 HS viruses were constructed for this study and were based on a modified pT7S3 (pT7S3-SpeI) that was modified to remove an SpeI site in the plasmid backbone such that it contained unique AflII and SpeI restriction enzyme sites in the regions encoding for L^pro^ and 2B, respectively. For each of the above infectious copy plasmids, DNA sequences covering the AflII site and the SpeI site and including the appropriate aa changes (as shown in Table 1, Table 2 and Table 3) were synthesised by Geneart. The synthesised DNA was initially cloned into pCR Blunt II-TOPO (Life Technologies, Paisley, UK) via blunt-end topisomerase cloning. The fragment containing the capsid region was recovered by double restriction digestion with AflII and SpeI and then ligated into similarly digested pT7S3-SpeI.

### 2.3. Virus Recovery

Infectious copy plasmids were linearised using HpaI and used as the template to synthesise full-length vRNA using an Ambion Megascript T7 kit (Life Technologies, Paisley, UK). Due to the presence of multiple HpaI sites, RNA was synthesised from the O1K-Asia WT and O1K-Asia KK plasmids without linearisation. The synthesised RNA (6.5 μg) was transfected into BHK-21 cells in 25 cm^2^ tissue culture flasks using Trans-IT mRNA transfection reagent (Mirus). Transfected BHK-21 cells were incubated at 37 °C for 24 hours (h) and then cell lysates were prepared by freeze-thawing. Viruses with wt capsids were subsequently passaged on BTY cells to minimise the chances of cell culture adaptation during recovery. All other viruses were passaged on adherent BHK-21 cells.

### 2.4. Viral Genome Sequencing

Clarified infected-cell lysates (0.5 mL) were added to 0.5 mL of trizol reagent (Life Technologies, Paisley, UK) and total RNA extracted as per the manufacturer’s instructions. The extracted RNA was converted to double-stranded cDNA, as described previously [39]. One nanogram of each cDNA sample (0.2 ng/μL) was used to prepare sequencing libraries using the Nextera XT DNA Sample Preparation Kit (Illumina, San Diego, CA, USA). Libraries were sequenced on a MiSeq using 300 cycle version 2 reagent cartridges (Illumina) to produce paired-end reads of approximately 150 bp each. Reads were assembled against an expected reference sequence using Seqman Ngen 14, and Seqman Pro 14 software (DNAstar, Madison, WI, USA) was used to view the line-ups and to generate a consensus sequence for each virus. Where additional non-synonymous mutations were found during virus recovery, the locations of aa changes were mapped onto the structure of an O_1_BFS FMDV pentamer (type O viruses; Protein Database ID:1FOD) or an A1061 FMDV pentamer (other serotypes; Protein Database ID:1ZBE).

### 2.5. Virus Titration (Plaque Assay)

Subconfluent BHK-21 or BTY cell monolayers in 6-well plates were incubated with serial dilutions of FMDV for 15 min at 37 °C. The cells were then overlaid with 4 mL of Eagle’s overlay (0.6% indubiose A37 (MP biomedicals, Santa Ana, CA, USA), 5% tryptone phosphate broth, 1% FCS in Eagle’s medium). After 48 h of incubation at 37 °C, the cells were fixed and stained with 4% formaldehyde/0.1% methylene blue.

### 2.6. Virus Purification

BHK-21 cells (O1K-O KK, O1K-O RK, O1K-O HS) or BTY cells (O1K-O WT) were infected with FMDV and freeze-thawed at the point of a complete CPE (cytopathic effect). The resulting lysate was clarified and the virus was precipitated by means of the addition of ammonium sulphate to give a 50% saturated solution. The precipitate was pelleted at 4800× *g* at 4 °C for 1 h and resuspended in phosphate-buffered saline (PBS) (pH 7.4) containing 1% IGEPAL CA-630. The virus was pelleted through a 30% sucrose cushion at 134,000× *g* (280,000 rpm) for 2.5 h, resuspended as described before, and purified by sedimentation through a 15–30% continuous sucrose gradient by centrifugation at 304,000× *g* (50,000 rpm) for 35 min. The purified virus was located by measuring the absorbance of gradient fractions at 260 nm using a spectrophotometer and quantified using the following formula: (OD260)/7.6 = mg/mL of virus.

### 2.7. Thermal Stability Assay

Thermal stability assays (PaSTRy assays) were performed using an MX3005 PCR machine (Agilent Technologies, Discot, UK) as described previously [40]. The assays were performed using 1 μg of purified virus and SYBR green-II dye (Invitrogen; final dilution 1:10,000) diluted to 100 μL in PBS buffer lacking divalent cations, and the sucrose content was kept constant between samples. The temperature was ramped from 25 °C to 94 °C in 0.5 °C increments with intervals of 20 seconds and fluorescence was read at each temperature (Excitation: 490 nm Emission: 516 nm). Data were visualised using MxPro version 4.10 software (Stratagene). Capsid dissociation, and hence the release of vRNA, was detected by the increases in fluorescence signal, and the temperature of RNA exposure (T_R_) was taken as the minimum of the negative first derivative of the fluorescence curve.

### 2.8. Adherent BHK-21 Cells: CPE and Infectious Yield Assays

Adherent BHK-21 cells were grown to 95% confluency in 96-well tissue culture plates. Wells were washed once with virus growth medium (VGM: normal cell culture medium with reduced (1%) serum) and infected with FMDV in a volume of 50 μL at a multiplicity of infection (MOI) of 0.01. After 1 h at 37 °C, the virus inoculum was replaced with 150 μL of VGM. The infection was continued at 37 °C and cell confluency in each well was quantified at 0.5 h intervals (10× objective, 4 images per well) for 50 h using the Incucyte S3 live cell analysis system (Sartorius, Gottingen, Germany) and Incucyte S3 2018B software using default software settings. To assess the rate of CPE, the average confluency of five replicate wells was plotted against time.

For virus yield experiments, BHK-21 cells in 96-well plates were infected (MOI = 0.01) and incubated at 37 °C until the cell confluency dropped below 80%. Cells were then freeze-thawed, cell supernatants and cell debris were separated by centrifugation, and the supernatant was transferred to separate Eppendorf tubes and stored at −80 °C. The infectious viral titre (using triplicate samples for each virus) was determined by a standard plaque assay on BHK-21 cells.

### 2.9. FMDV Growth in Suspension BHK-21 Cell Bioreactors

A BHK-21 suspension culture in log growth phase was transferred to 400 mL mini-bioreactors (AppliKon Biotechnology, Delft, The Netherlands) at a density of ~2 × 10^6^ cells/mL. Cells were settled to the bottom of the reactor at 5 °C and the medium (GMEM + 5%ABS) was replaced with GMEM without ABS. After resuspension of the cells, the culture was infected with the relevant virus at an MOI of 0.001 under pH and dO2 control (pH 7.3, 40% dO2) at 37 °C and with continuous stirring at 400 rpm. Virus stocks used for infection were made on the same batch of primary pig kidney cells and were sequenced to check for aa changes in the capsid. At 16 h, 19 h, 22 h and 40 h post-infection, samples were taken, of which 15 mL was directly frozen at −80 °C (harvest sample) and 15 mL was clarified by centrifugation for 20 min at 2585× *g* (3400 rpm) and then stored (clarified samples) to mimic centrifugation downstream processing steps in industrial operations. Yield data from suspension BHK cells presented in this study were obtained using the “clarified” samples. To identify dead cells, live cell counts were carried out using a counting chamber (Fuchs Rosenthal) and cells were diluted in PBS plus Trypan Blue (0.4%) (Life technologies, Paisley, UK). Cell counts were used to calculate CPE using the following formula:

$$\mathrm{Cytopathic}\;\mathrm{Effect}\;(\mathrm{CPE}\;\%),\;CPE\left(t\right)=(1-\frac{\left[VCD\;at\;t\;hours\;pi\right]}{\left[VCD\;before\;infection\right]})\times 100$$

VCD = viable cell density

Suspension culture experiments were performed in triplicate.

For chimeric viruses with type A and Asia-1 capsids, antigen from a single batch was used for antiserum generation.

### 2.10. Quantitation of 146S Particles from Bioreactor Cultures by ELISA

The yield of 146S particles for FMDV with type A and type O capsids was determined by the enzyme-linked immunosorbent assay (ELISA) [41]. Briefly, 96-well plates (Corning) were coated overnight at 4 °C with 100 µL (0.05 µg/mL) per well of 12S-specific M3 single-domain antibody fragments (VHH). The wells were washed and sequentially incubated for 1 h at room temperature (RT) with twofold serial dilutions of unheated or heated (2 h at 56 °C to dissociate 146S in to 12S) FMDV samples, biotinylated M3 (0.1 µg/mL), and 1:10,000 streptavidin-HRP (Jackson ImmunoResearch), with washing between incubations. After a final washing step, 100 μL/well TMB (3,3′,5,5′-Tetramethylbenzidine) was added and the reaction was stopped after ten minutes using 0.5 M H_2_SO_4_. The absorbance was measured at 450 nm and the 12S content was calculated using a standard curve generated using 12S particles of the FMDV O_1_Manisa reference strain. The difference between the 12S content of unheated and heated samples (to dissociate 146S into 12S) is a measure for the amount of 146S particles present. ELISAs were only valid when the internal control differed by less than two SDs from the trend. For each 400 mL culture experiment, ELISAs were performed twice.

### 2.11. UV-Peak for 146S Quantification

The yield of 146S particles for FMDV with Asia-1 and SAT2 capsids was determined by sucrose density gradients as described previously [42]. Briefly, a 2 mL antigen sample was slowly added into 10 mL premade discontinuous 15–45% sucrose gradient solutions. Gradients were centrifuged at 48,700× *g* (16,500 rpm) at 5 °C for 16 h in an ultracentrifuge (Optima XE-90; Beckman Coulter, Brea, CA, USA). The centrifuged solution was pumped through a UV detector (LKB-Bromma 2238 UVICORD S-II; Cytiva, Marlborough, MA, USA) at an adsorption of 254 nm. Based on the area under the curve of the UV peak, the 146S antigenic mass could be calculated. All assays were performed in duplicate, and assays were only valid when a known reference differed by less than two SDs from the trend.

### 2.12. Antiserum Production

Antisera for the virus neutralisation tests (VNTs) were raised against the chimeric viruses with type A and Asia-1 capsid using 13-week-old healthy SPF White New Zealand rabbits. Immunisation in rabbits was approved by the ethical committee of Wageningen University and Research under project number 2016.D-0062.13 and was carried out in the Veterinary BSL-4 facilities at Wageningen Bioveterinary Research, Lelystad, The Netherlands.

In order to raise the antisera, vaccines were prepared by infecting 400 mL suspension cultures of BHK-21 cells with O1K-A WT, O1K-A KK, O1K-A RK, O1K-Asia WT, O1K-AsiaI KK, or O1K-Asia HS virus. Virus cultures were inactivated by the addition of 0.01 M Binary ethylenimine (BEI) for 24 h at RT. BEI was neutralised by the addition of 0.5% sodium thiosulphate for 1 h at RT. Antigens were concentrated by means of precipitation with 7.5% polyethylene glycol for 16 h at 4 °C. After centrifugation at 2500× *g* for 45 min at 4 °C, the pellet was resuspended in Tris/KCl (20 mM Tris, 300 mM KCl, pH 7.5) buffer and the antigen concentration was determined using the UV peak.

Groups of four rabbits were subcutaneously vaccinated with 0.5 mL of 20 μg/mL (O1K-A WT, O1K-A KK, O1K-A RK) or 15 μg/mL (O1K-Asia WT, O1K-AsiaI KK or O1K-Asia HS) antigens diluted 1:1 in Tris/KCl and Montanide ISA50V2 (Seppic). After 21 days, the rabbits were booster vaccinated using the same method. At 42 days post-vaccination, the rabbits were sacrificed and serum was retrieved from the blood by means of centrifugation and stored at −20 °C until use in VNTs.

### 2.13. Virus Neutralisation Tests Using IBRS-2 Cells

Virus stocks used in the VNT were grown on IBRS-2 monolayers and were titrated in triplicate using the same cells. Experimental serum samples obtained by vaccination of rabbits using viruses generated in this study (see “Antiserum production”) were inactivated at 56 °C for 0.5 h before testing. Virus neutralisation tests were performed according to the protocol recommended by the World Organisation for Animal Health [43,44]. Neat serum stocks were initially diluted 1:8 and then in two-fold dilutions for the tests. For each test, a 100 TCID50 of virus was used in a total volume of 50 μL. Neutralising antibody titres, calculated by the Spearman–Karber method, were expressed as the last dilution of serum that neutralises 50% of the virus.

### 2.14. Virus Neutralisation Tests Using BTY Cells

Virus stocks used in the VNT were grown on BTY monolayers and were titrated in triplicate using the same cells. Anti-Asia-1, -type O, -type A, and -SAT-2 antisera were obtained from the World Reference Laboratory at Pirbright. Virus samples and sera were diluted in reduced serum (1%) BTY cell culture medium. Sera were diluted to an initial dilution of 1/8 or 1/16, diluted two-fold six subsequent times in duplicate, and 70 μL of the serum dilutions were added per well of a 96-well flat bottom plate. Diluted viruses (70 μL) were added to the wells with the serum dilutions, such that each well contained 200 TCID50 per 100 μL. The mixtures were incubated for 1 h at RT. BTY cells grown to confluency in a separate 96-well plate were washed once with reduced serum BTY medium, and 50 μL of BTY medium was added to each well. The antibody/virus mixtures (100 μL) were transferred to the equivalent well of the 96-well plate containing BTY cells. Plates were then incubated for 3 days at 37 °C and 5% CO_2_, in a humidified atmosphere, and then fixed and read as above for IBRS-2 cells.

### 2.15. Statistical Analysis

Statistical significance between groups was assessed via an unpaired two-tailed *t*-test. A *p*-value of <0.05 was considered statistically significant. Data distribution was analysed via the Ryan Joiner normality test using Minitab 10 software prior to the *t*-test.

## 3. Results

### 3.1. Design of Chimeric Viruses

The chimeric FMDVs used in this study are based on the FMDV O1K infectious copy plasmid, pT7S3, and were made by capsid switching (see Section 2). The capsids used to generate viruses with wt capsids, O1K-A WT, O1K-Asia WT, O1K-O WT, and O1K-SAT2 WT, were derived from the A-Turkey/2/2006, Asia-1/Bar/9/2009, OUKG/35/2001 and SAT2/EGY/9/2012 field strains, respectively. We also generated variants of the above viruses with capsid aa substitutions that are associated with cell culture adaptation. The changes were introduced at two sites: (i) the icosahedral 5-fold symmetry axes and (ii) the surface depression that forms the canonical HS binding site.

In the O1K-A WT and O1K-Asia WT viruses, the residues at VP1 109 and 110 are K and Q, respectively, whereas for O1K-O WT, they are K and A. We have previously generated variants of the above viruses with a single K substitution at VP1 110 [32] (here called O1K-A KK, O1K-Asia KK, and O1K-O KK; note that KK indicates the presence of a K residue at both VP1 109 and 110). As a result of this substitution, the O1K-A KK, O1K-Asia KK, and O1K-O KK viruses acquired an ability to infect CHO cells (which lack the integrin receptors utilised by FMDV field strains), indicating that they initiate infection using non-integrin receptors. We also previously described a chimeric O1K-A virus with R at VP1 109 and K at VP1 110 (here called O1K-A RK) and confirmed that this virus could also infect CHO cells. For the current study, we generated variants of the O1K-Asia WT and O1K-O WT viruses that also have RK at VP1 109-110 (called O1K-Asia RK and O1K-O RK). These viruses are described in Table 1.

For SAT viruses, aa changes that map to the icosahedral 5-fold symmetry axes have also been associated with cell culture adaptation and an acquired ability to infect CHO cells [25,26]. These changes often introduce positively charged residues and are commonly seen at VP1 83–85 and/or 110–112. Therefore, we also generated variants of O1K-SAT2 WT with positively charged aa substitutions at these sites. In the SAT2/EGY/9/2012 field virus, VP1 83–85 has the sequence DHT, which we changed to KHR (generating O1K-SAT2 KHR; see Table 2). The sequence of VP1 110–112 is KGG, and this was changed to KGR or KKR (generating O1K-SAT2 KGR and O1K-SAT2 KKR, respectively).

The crystal structures of FMDV O1BFS and A1061 bound to heparin show that the canonical HS binding site is located in a shallow depression on the outer capsid surfaces that is formed by all three outer capsid proteins (VP1–VP3). The base of the HS binding site is formed by VP3 84–88, and the walls by VP2 134–138, VP3 55–60, and VP1 195–197 (O1BFS) or 193–195 (A1061). For both O1BFS and A1061, the main heparin contact residues are VP2-134 (K in O1BFS and T in A1061) and -135 (R in both O1BFS and A1061), VP3-56 (R in both O1BFS and A1061), and VP1-195H (in O1BFS) or 193K (in A1061). Most of the residues that form the HS binding site on O1BFS are conserved in the O1K-O WT virus, and only two aa changes were required to generate a HS binding site with an aa sequence that was an exact match for O1BFS. These changes were made to create a virus, O1K-O HS (Table 3). Similarly, most of the residues that form the HS binding site on A1061 are conserved in the O1K-A WT virus and only four aa changes were required to generate a HS binding site that matched A1061 (see Table 3, O1K-A HS). Although viruses with aa changes at the depression that forms the HS binding have not been seen for Asia 1 and SAT2 viruses, for O1K-Asia WT and O1K-SAT2 WT, we also attempted to create a HS binding site. We based the aa changes on the sequence of A1061 so as to minimise the number of substitutions; however, seven residue changes were required to generate O1K-Asia HS, and ten to generate O1K-SAT2 (see Table 3).

### 3.2. Virus Recovery

The chimeric viruses were recovered from BHK-21 cells transfected with vRNA in vitro transcribed from the appropriate IC plasmids. As they are dependent on integrins to initiate infection, viruses with wt, unmodified capsids were subsequently passaged on BTY cells, and all four viruses with wt capsids were readily recovered. After transfection, viruses with aa changes in the capsid proteins were passaged on BHK-21 cells. Of the nine viruses with changes at the 5-fold symmetry axes, only six (Table 1 and Table 2) could be recovered using the above approach, as the O1K-Asia RK, O1K-SAT2 KHR, and O1K-SAT2 KGR viruses did not show CPE on BHK-21 cell passage. Of the four viruses with changes at the canonical HS binding site, only two (O1K-O HS and O1K-Asia HS) were viable, as the O1K-A HS and O1K-SAT2 HS viruses could not be recovered. For the five viruses (O1K-Asia RK, O1K-SAT2 KHR, O1K-SAT2 KGR, O1K-A HS and O1K-SAT2 HS) that could not be recovered on BHK-21 cells, we also attempted to recover infectious virus using BTY cells for the post-transfection passage steps. This showed that four of the viruses were non-viable, as only the O1K-SAT2-KGR virus caused CPE when passaged on BTY cells. The reasons why these viruses could not be recovered (on BHK-21 and/or BTY cells) are currently unknown (see Section 4) and were not investigated further.

### 3.3. Characterisation of the Recovered Viruses

**Figure 1:**
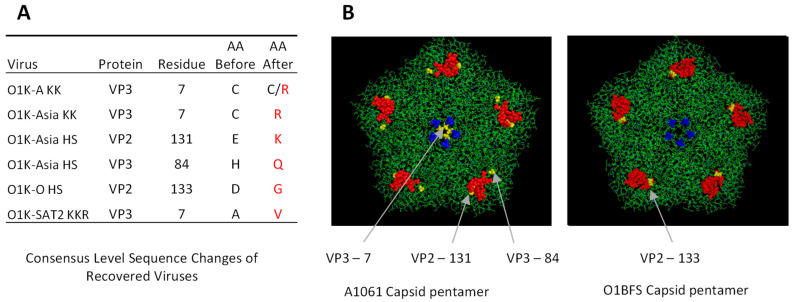
Identity and location of consensus level amino acid changes observed during virus recovery. The additional amino acid changes in the capsid arising during virus recovery are shown in the left panel (Panel (**A**)). The expected (AA before) and actual (AA after; highlighted in red where different to AA before) residues of the recovered viruses (at BHK P4) are shown. The right panel (**B**) shows the positions of the additional changes (shown in yellow) relative to the introduced changes at the 5-fold symmetry axes (VP1-109 and -110 are shown in blue) or at the canonical HS binding site (red). The locations of the additional changes are shown on pentamer models of O1BFS (for changes observed in type O (reference 1FOD)), or A1061 (for the other viruses (reference 1ZBE)).

Whole-genome sequencing (using a non-PCR-based Miseq protocol [39]) at passage 4 (p4) showed that the recovered viruses with a wt capsid and three of the viruses (O1K-A RK, O1K-O KK, O1K-O RK) with capsid aa substitutions faithfully retained the sequence of the input RNA. However, five of the recovered viruses with introduced aa substitutions acquired additional aa changes in the capsid during virus recovery (Figure 1). In each case, the additional changes were located close to the site of the introduced aa substitution(s). Three of the viruses (O1K-A KK, O1K-Asia KK, and SAT-2 KKR) had an additional aa change (VP3-7) that lies close to the icosahedral 5-fold symmetry axes, whereas O1K-O HS and O1K-Asia HS had additional changes at VP2-133 (O1K-O HS), or at VP2-131 and VP3-84 (O1K-Asia HS), which lie close to the canonical HS binding site. Interestingly, in keeping with changes seen when FMDV is adapted to cell culture, some of the changes resulted in a net gain of positively charged residues.

Cell culture adaptation of FMDV often results in the ability to use novel non-integrin receptors and to infect CHO cells. To ascertain if the recovered viruses could use non-integrin receptors, we investigated if they caused CPE on CHO cells. BTY cells were included as a control as all of the recovered viruses retain the RGD motif on VP1 and are expected to infect BTY cells using integrin αvβ6 as the receptor. As expected, while none of the viruses with a wt capsid caused CPE on CHO cells, they all caused extensive CPE in BTY cells. Furthermore, all of the recovered viruses with capsid aa substitutions caused CPE in CHO and BTY cells. These results show that the recovered viruses with aa changes in the capsid can use a non-integrin receptor to initiate infection.

We also determined the relative infectivity for BHK-21 and BTY cells of the recovered viruses (at p4; BHK-p4 or BTY-p4). Table 4 shows the titre of the recovered viruses on each cell type. The titre values on BHK-21 and BTY cells cannot be compared directly since the titres depend on the time of harvest, and the cell line used for virus propagation, both of which differed for the different viruses. Therefore, we used the ratio of the titre on BTY and BHK-21 cells, as this better reflects the relative efficiency of infection. A high BTY/BHK ratio indicates a higher infectivity for BTY cells compared to BHK-21 cells. As expected, viruses with unmodified wt capsids had relatively high BTY/BHK ratios indicating that they could infect BTY cells with a greater efficiency than BHK-21 cells. This was especially apparent for the O1K-O WT and O1K-SAT2 WT viruses as the BTY/BHK ratio for these viruses were very high (>2000 and >20,000, respectively), which reflects the low titres of these viruses on BHK-21 cells. The BTY/BHK titre ratio for the O1K-A WT and O1K-Asia WT viruses were lower (18 and 11.5, respectively) than for O1K-O WT and O1K-SAT2 WT, as they generated a higher titre on BHK-21 cells. Notably, all of the viruses with capsid aa substitutions generated a lower BTY/BHK ratio than the corresponding virus with the wt capsid. For O1K-O viruses (HS, KK and RK) and O1K-SAT2 KKR, the reduction in the BTY/BHK ratio was most dramatic, as for these viruses, the efficiency of infection of BHK-21 cells was significantly improved. These data indicate that, for each chimeric virus, the introduced aa substitutions enhance the infection of BHK-21 cells, most likely due to altered receptor usage, as evidenced by the ability to infect CHO cells.

### 3.4. Effect of the Introduced Changes on Capsid Stability

**Figure 2:**
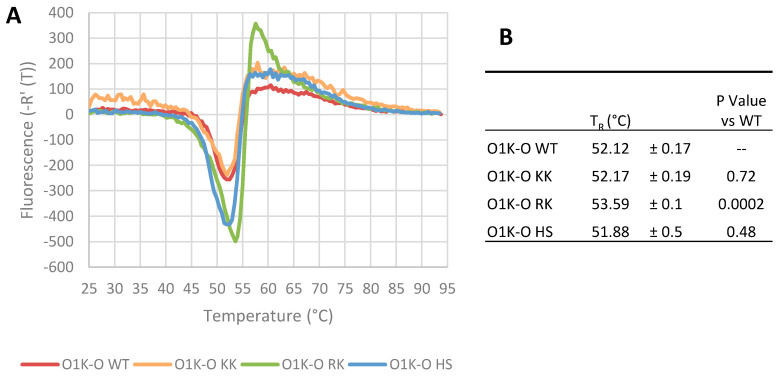
Thermal stability of the recovered type O viruses. The thermal stability of the recovered type O viruses was determined using the pASTRY assay. Gradient purified virus (1 μg) was mixed with Sybr Green II RNA binding dye, and fluorescence was read at 0.5 °C intervals for 20 s at each temperature. The experiment was repeated three times with triplicate samples. Panel (**A**) shows representative traces of the inverse first differential of fluorescence against temperature for each virus. Panel (**B**) shows the mean ± SD of the T_R_ value for each virus. The T_R_ value is the temperature of the RNA genome’s exposure to dye as calculated from the temperature at which the inverse first differential fluorescence curve is at its minimum, and a higher T_R_ indicates increased particle stability. The *p*-values for each of the modified viruses (paired with O1K-O WT) were calculated by unpaired *t*-test (two tailed).

Amino acid changes in the capsid could influence virus particle stability, which could negatively impact infectivity and vaccine efficacy, as intact 146S particles produce a much greater protective immune response than dissociated 12S pentamers [45]. To test the effects of the aa changes on particle stability, we used the viruses with type O capsids, since a greater number of viruses with modified capsids were recovered, and the type O viruses are relatively unstable when subjected to heat compared to other serotypes [46]. Viruses were purified by sucrose density gradient ultracentrifugation and particle stability assessed using a pASTRY assay (Particle Stability Thermal Release Assay) [40]). In this assay, viruses are exposed to increasing temperature until particle stability is compromised, exposing the vRNA genome, which can then be detected using an RNA-binding fluorescent dye. Thus, the temperature of genome release (T_R_) (defined as the inflection point of a differential curve of fluorescence intensity against temperature) reflects particle stability. Representative traces from these assays are shown in Figure 2A, and the calculated T_R_ values in Figure 2B show that the O1K-O KK and O1K-O HS viruses had a similar stability threshold to the wt virus, whereas the O1K-O RK virus appears to show a small but statistically significant increase in thermal stability.

### 3.5. Effect of the Introduced Changes on the Antigenic Properties of the Recovered Viruses

**Figure 3:**
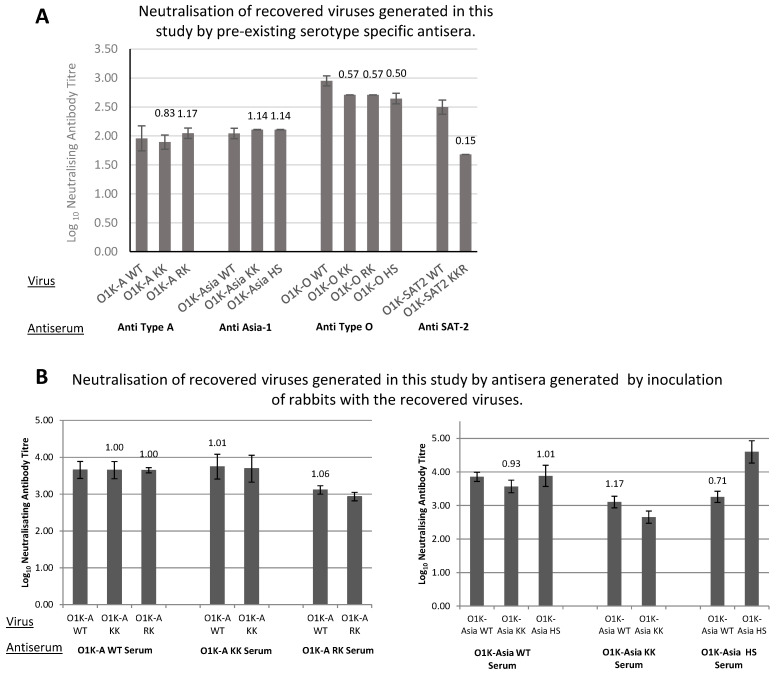
Antigenic properties of the recovered viruses. (**A**) Virus neutralisation tests were carried out in BTY cells using 200TCID50 of the indicated virus/well and doubling dilutions of the indicated pre-existing antisera generated against a related virus of the same serotype obtained from the World Reference Laboratory, Pirbright, UK. Data are shown as the log10 (mean ± SD from triplicate experiments) of the neutralising antibody titre. The R1 values for each virus (determined against the virus with the WT capsid) are shown above the bars. (**B**) Virus neutralisation tests were carried out in IBRS-2 cells, using 100TCID50 of the indicated virus/well and doubling dilutions of the indicated antiserum generated against the recovered viruses in rabbits. The bars show data for individual viruses that were neutralised by the indicated antiserum (serum). Data are shown as the log10 of the neutralising antibody titre and presented as the mean ± SD of three experiments using the mean titre from four separate rabbit sera. The R1 values for each virus (heterologous virus against homologous virus) are shown above the bars.

It is also possible that aa changes introduced into the capsid could affect the antigenicity of the viruses. Therefore, we compared the antigenic properties of viruses with wt or modified capsids using virus neutralisation tests (VNTs). Initially, we used existing cattle sera generated against viruses (or vaccines) that are closely related to the field strains from which the capsids used to construct the chimeric viruses were derived. These VNTs were carried out using BTY cells, as some of the viruses (particularly O1K-O WT and O1K- SAT2 WT) grow very poorly on the cell lines typically used for such assays (BHK-21 or IBRS-2). Briefly, the viruses were mixed with doubling dilutions of neutralising antiserum, inoculated onto BTY cells, and the antibody neutralisation titre was measured, expressed as the dilution of antibodies that gave 50% protection against CPE. Figure 3A shows the antibody neutralisation titres (expressed as log_10_ of the dilution of antibodies that gave 50% protection of BTY cells against CPE) and that all of the O, A, and Asia-1 viruses with modified capsids had similar antibody neutralisation titres to the corresponding viruses with a wt capsid. The antigenic match between two viruses can be expressed as a ratio, referred to as R1 (here, R1 is the antibody neutralisation titre for a modified virus divided by that for the virus with the wt capsid). For vaccine matching purposes, an R1 cut-off value of 0.3 or above is used to indicate a good vaccine match according to the OIE manual [43]. For the type A, Asia-1, and O viruses with modified capsids, the R1 values were all above 0.3, suggesting that the introduced aa changes are unlikely to affect the principal antigenic neutralisation sites on the capsid. In contrast, the neutralisation titre for O1K-SAT2 KKR was notably lower (R1 = 0.15) than that for O1K-SAT2 WT. However, it remains to be determined if this would be a problem for vaccine efficacy in vivo (see Section 4).

The above results suggest that (with the possible exception of O1K-SAT2 KKR) the antigenic integrity of the viruses with aa changes in their capsid proteins are unaltered. However, the data were obtained using pre-existing sera raised against closely related viruses. Therefore, we sought to further investigate whether the capsid sequence changes affected the antigenic properties of the viruses. To achieve this, we generated vaccine material for the wt and modified type A and Asia-1 viruses (i.e., O1K-A WT, O1K-A KK, O1K-A RK, O1K-Asia WT, O1K-Asia KK, O1K-Asia HS). The type O and SAT2 viruses could not be used, as O1K-O WT and O1K-SAT2 WT show poor growth in conventional cell lines, which made the production of vaccine material for these viruses difficult. The vaccines were used to inoculate rabbits, and serum was collected at 42 days post-inoculation. Virus neutralisation tests were repeated using the generated antisera on IBRS-2 cells, as O1K-A WT and O1K-Asia WT viruses showed good infection on these cells. For each antiserum, VNTs were carried out using the virus used to generate the antiserum (homologous virus) and all of the other viruses of the same serotype (heterologous viruses). As shown in Figure 3B, all the antisera demonstrated high neutralisation titres when tested against both homologous and heterologous viruses of the same serotype. All R1 values (in this case, the antibody titre for heterologous viruses divided by the titre for the homologous virus) ranged between 0.7 and 1.1, which is well above the 0.3 cutoff. Importantly, antisera generated using viruses carrying altered capsids generated good neutralisation titres when tested in VNT assays using viruses carrying wt capsids (e.g., the O1K-A KK antiserum generated a neutralisation titre approaching 4 when tested against both the heterologous O1K-A WT virus and homologous O1K-A KK virus, with an R1 value of 1.01). Together, the above results indicate that the capsid changes introduced at either the icosahedral 5-fold symmetry axes or at the canonical HS binding site of A-Turkey/2/2006, Asia-1/Bar/9/2009 or OUKG/35/2001 are unlikely to be detrimental to the production of neutralising antibodies against the field viruses.

### 3.6. Growth of the Modified Viruses in Adherent BHK-21 Cells

**Figure 4:**
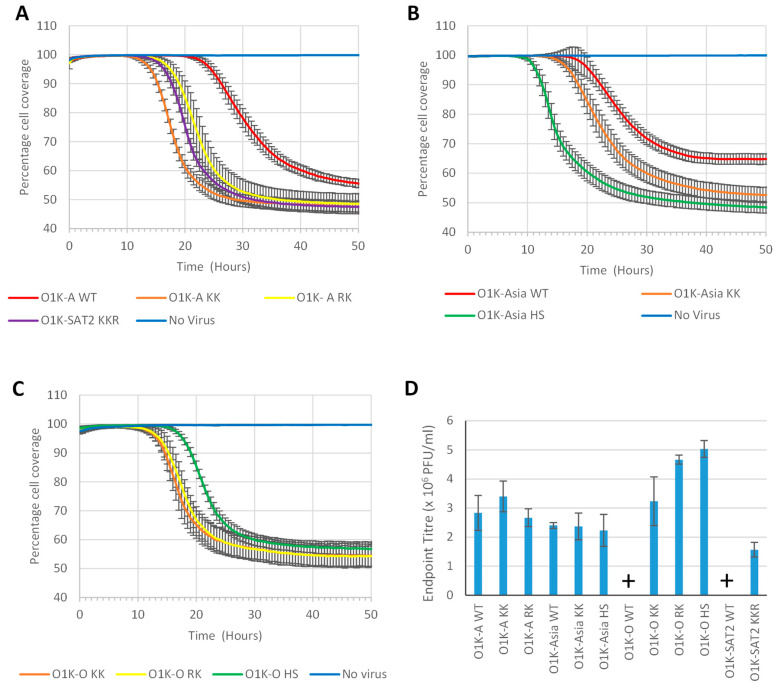
Rate of CPE in adherent BHK cells. Confluent monolayers of adherent BHK cells in a 96-well plate were infected with the indicated virus (Panels (**A**–**C**): MOI of 0.01) and the progression of CPE (as measured by the reduction in percentage cell coverage) monitored over time. Data are shown as the mean ± SD for five replicate wells from one experiment representative of two that gave similar results. (**D**) Triplicate BHK monolayers in 96-well plates were infected (MOI of 0.01) with the indicated virus. When cell coverage dropped below 80% (as determined in panels **A**–**C**), the infections were stopped by freezing the cells. Virus titres were quantified by plaque assay on BHK cells, and the mean ± SD of triplicate samples are shown. Note: For the O1K-O WT and O1K-SAT2 WT viruses, an MOI of 0.01 could not be achieved, and thus they are not included in (**A**–**C**), and are marked with a “+” symbol in (**D**).

Next, we investigated the growth rate (development of CPE) and virus yield (endpoint virus titre) of the recovered viruses in adherent BHK-21 cells. Confluent BHK-21 cell monolayers were infected with FMDV (MOI of 0.01) and transmitted light images taken every 0.5 h using an Incucyte imaging system and analysed to assess the percentage cell coverage. As CPE develops, cells detach from the plate, thereby reducing the proportion of the area of cell coverage. Figure 4A shows that for the type A viruses, the KK variant caused CPE at a faster rate than the RK variant, but both caused CPE faster than O1K-A WT. For the Asia-1 viruses, the HS variant caused more rapid CPE than the KK variant, and both caused CPE faster than O1K-Asia WT (Figure 4B). We could not include the O1K-O WT and O1K-SAT2 WT viruses in these experiments, as they infect BHK-21 cells poorly (see Table 4), and the desired MOI of 0.01 could not be achieved. However, we did include the type O viruses (Figure 4C) with modified capsids. This showed that the viruses with changes at the icosahedral 5-fold symmetry axes (O1K-O RK and O1K-O KK) caused CPE slightly faster than the HS variant, and at a similar rate to O1K-A RK and O1K-A KK (Figure 4A). Similarly, the O1K-SAT2 KKR variant (Figure 4A) caused CPE at a similar rate as O1K-A KK and O1K-A RK. These results show that for each of the recovered viruses, the engineered changes allow for more rapid virus spread through adherent BHK-21 cells.

To more accurately determine virus yields, BHK-21 cells were infected as above (MOI 0.01) and the cells were frozen at the point of maximum CPE (as determined by the analysis described above) and virus titres were assessed by plaque assays on BHK-21 cells. Figure 4D shows that for the type A viruses, the RK and KK variants had similar endpoint titres to O1K-A WT. Similarly, for the Asia-1 viruses, the KK and HS variants reached similar titres to O1K-Asia WT. As mentioned above, an MOI of 0.01 could not be achieved for the O1K-O WT and O1K-SAT2 WT viruses and endpoint titres for these viruses could both not be determined. However, all of the type O viruses with introduced aa changes and the O1K-SAT2 KKR virus showed similar virus yields to the other viruses with the same modifications. Thus, the aa substitutions that were introduced into the chimeric viruses with type O or SAT-2 capsids greatly improved virus yield. The above results show that for viruses with a wt capsid (i.e., O1K-A WT, and O1K-Asia WT) that infect BHK-21 cells relatively well, the introduction of aa changes at icosahedral 5-fold symmetry axes or at the canonical HS binding site appears to increase the rate of CPE, but does not significantly improve virus yield, whereas when the changes are introduced into viruses (i.e., O1K-O WT and O1K-SAT2 WT) with a wt capsid that show poor infection of BHK-21 cells, both the rate of CPE and virus yield are greatly enhanced.

### 3.7. Growth of the Modified Viruses in Suspension BHK-21 Cells

**Figure 5:**
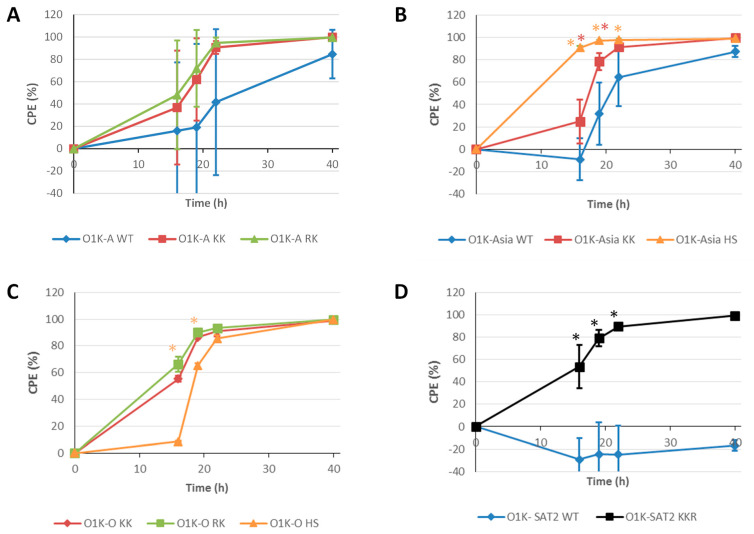
Rate of CPE in suspension BHK cells. Suspension BHK cells in 400 mL minibioreactors were infected with the indicated viruses at an MOI of 0.001. Cells were collected at 16 h, 19 h, 22 h, and 40 h post infection and the cell viability rate was determined by counting viable cells. Each data point represents the mean ± SD from triplicate experiments. Note: For the O1K-O WT virus, an MOI of 0.001 could not be achieved, and thus it was not included in the experiment. Asterisks indicate that the CPE value at that timepoint is significantly different (as determined by *t*-test) for the virus indicated by the asterisk colour from the value for the virus carrying WT capsid (panels (**A**,**B**,**D**)) or the value for the O1K-KK virus (Panel (**C**)).

As vaccine viruses (146S particles) are generated using suspension BHK-21 cells, we also investigated the development of CPE and the yield of intact 146S antigen using suspension BHK-21 cells (Figure 5). Suspension BHK-21 cells in 400 mL bioreactors were infected at an MOI of 0.001 and CPE was followed over time (16 h, 19 h, 22 h and 40 h post-infection) by counting viable cells. For the viruses with type A capsids, the RK and KK variants appeared to cause faster CPE than O1K-A WT; however, the variability between replicates was high and the data did not support statistical significance (Figure 5A). For the viruses with Asia-1 capsids, the viruses with capsid aa changes (O1K-Asia KK and O1K-Asia HS) also caused faster CPE relative to O1K-Asia WT (Figure 5B). In this case, the rate of CPE for the O1K-Asia HS virus showed a statistically significant increase compared to the other two viruses (O1K-Asia KK and O1K-Asia WT). Due to its poor infectivity of BHK-21 cells, we could not prepare sufficient amounts of the O1K-O WT virus for this experiment. Nevertheless, all three viruses derived from O1K-O WT with aa changes in the capsid caused significant CPE (Figure 5C), with a similar rate to the A and Asia-1 viruses with modified capsids. In addition, the O1K-SAT2 KKR virus also caused CPE at a much faster rate than O1K-SAT2 WT (Figure 5D). These results show that all of the recovered viruses with aa changes engineered into the capsid appear to cause more rapid CPE in suspension BHK-21 cells than the viruses with the corresponding wt capsid.

**Figure 6:**
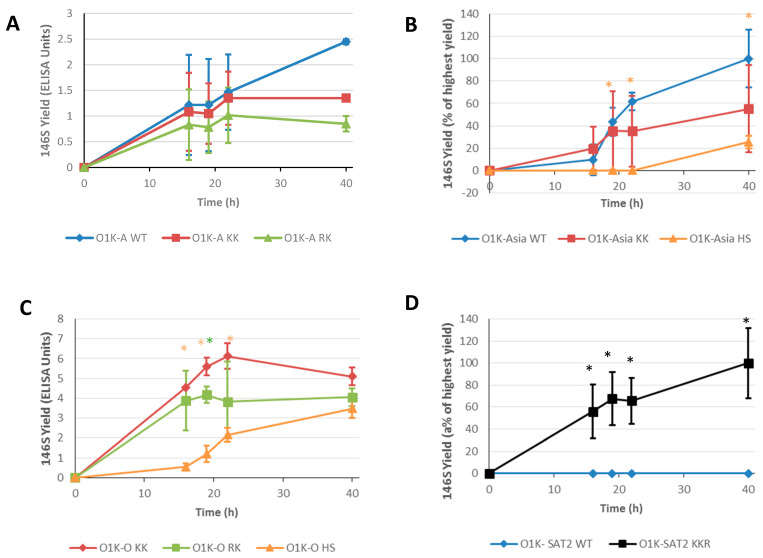
146S particle yields generated in suspension BHK cells. Suspension BHK cells in 400 mL minibioreactors were infected with the indicated viruses at an MOI of 0.001. Samples were collected at 16 h, 19 h, 22 h, and 40 h post infection. Yields of 146S particles were calculated by ELISA using the camelid VHH M3 (Panel (**A**,**C**)) or sucrose density gradient ultracentrifugation followed by UV analysis (Panels (**B**,**D**)). Data are shown as the mean ± SD from triplicate experiments. Note: For the O1K-O WT virus, an MOI of 0.001 could not be achieved, and thus it was not included in the experiment. Asterisks * indicate that the 146S yield at that timepoint is significantly different (as determined by *t*-test) for the virus indicated by the asterisk colour, from the 146S yield for the virus carrying WT capsid (panels (**A**,**B**,**D**)) or the value for the O1K-KK virus (Panel (**C**)).

A vaccine’s efficacy is greatest when it has a high content of intact 146S virus particles. Therefore, we also determined the yield of 146S particles generated during infection of suspension BHK-21 cells in a bioreactor (Figure 6). For type A and type O viruses, the amounts of 146S particles were quantified by ELISA using the camelid VHH M3 antibody, whereas for the SAT-2 and Asia-1 viruses, we used sedimentation sucrose density gradients. For the type A and Asia-1 viruses (Figure 6A and Figure 6B, respectively), most of the viruses carrying introduced aa changes did not show differences in 146S yield relative to the corresponding virus with the wt capsid. The only exception was the O1K-Asia HS virus, which showed a statistically significantly lower yield relative to O1K-Asia WT. For the type O viruses (Figure 6C), O1K-O WT was not included due to its poor infectivity of BHK-21 cells as mentioned; thus, the 146S yields obtained for the O1K-O KK, O1K-O RK, and O1K-O HS viruses represent a significant improvement over O1K-O WT. Interestingly, as seen for O1K-Asia HS, the HS variant (O1K-O HS) gave a lower yield than the variants with changes at the icosahedral 5-fold symmetry axes (O1K-O KK, O1K-O RK). Strikingly, for the SAT-2 viruses (Figure 6D), the virus with a wt capsid (O1K-SAT2 WT) gave essentially no detectable 146S yield, and hence the O1K-SAT2 KKR virus shows a greatly enhanced yield.

Collectively, the above data show that for the chimeric viruses with a wt capsid that can readily infect adherent and suspension BHK-21 cells (O1K-A WT and O1K-Asia WT), the introduction of aa changes at the icosahedral 5-fold symmetry axes or at the canonical HS binding site increases the rate of CPE, but does not appear to significantly improve the yield of 146S particles. In contrast, when aa changes are introduced into viruses with a wt capsid that shows poor infection of BHK-21 cells (O1K-O WT and O1K-SAT2 WT), both the rate of CPE and 146S yield are greatly enhanced.

## 4. Discussion

The production of a new vaccine to combat an emerging novel variant of FMDV may require the adaptation of the outbreak virus to BHK cells. This can be time consuming, as field viruses often grow poorly in the suspension BHK cell lines used for vaccine manufacture. The adaptation process is driven, in part, by the availability of integrin receptors that are used by FMDV field viruses to enter cells, and it selects variants with capsid aa changes that allow use of novel cell attachment receptors. Interestingly, even for viruses of the same FMDV serotype, this process does not always involve aa changes at the same site on the capsid or result in the ability to use a common alternative receptor [20,24,25,26,27,28,29,32,33,34,35,47]. Furthermore, changes in the capsid may be different when the same FMDV strain is adapted to adherent or suspension BHK cell culture systems [48,49].

Previously, we [32,47] and others [21,26,27,50] have generated chimeric viruses that combine the capsid proteins of FMDV field strains with the non-structural proteins (nsps) of cell-culture-adapted viruses. Such chimeric viruses have proven useful to study the aa changes in the capsid proteins that allow for integrin-independent infection of cultured cells, such as BHK-21 and CHO cells. Furthermore, it has been proposed that such viruses could be engineered to include aa changes in the capsid proteins that allow for infection of BHK-21 cells, as a means to bypass the need for cell culture adaptation, especially for field viruses when adaptation proves intractable. In the current study, we used four chimeric viruses (O1K-A WT, O1K-Asia WT, O1K-O WT and O1K-SAT2 WT) with wt capsids derived from the FMDV field strains A-Turkey/2/2006, Asia-1/Bar/9/2009, OUKG/35/2001, and SAT2/EGY/9/2012. All four could be readily recovered using BTY cells, and two (O1K-A WT and O1K-Asia WT) could readily infect BHK-21 cells without the need to make additional aa substitutions. In contrast, the other two viruses (O1K-O WT and O1K-SAT2 WT) were poorly infectious for BHK-21 cells. A possible explanation for this is that whilst viruses with wt capsids are able to use the αvβ6 integrin, the preferred receptor used by field strains and expressed on BTY cells [51], they may have different abilities to utilise the αvβ3 integrin receptor expressed on BHK-21 cells [7]. This is consistent with previous work reporting that type O viruses use αvβ3 much less efficiently than type A viruses [52]. In an attempt to bypass the need for cell culture adaptation, we also generated variants of the above chimeric viruses with aa changes in the capsids and characterised their growth in adherent and suspension BHK-21 cells. We targeted two sites on the capsid: the canonical HS binding site (as identified on FMDV O1BFS and A1061) and the icosahedral, 5-fold symmetry axes of the capsid. In addition, the different abilities of the viruses with wt capsids to infect BHK-21 cells allowed us to investigate the effects of introducing aa substitutions into viruses that could or could not already infect BHK-21 cells.

For the chimeric viruses with capsids derived from A-Turkey/2/2006 (O1K-A KK and O1K-A RK), Asia-1/Bar/9/2009 (O1K-Asia KK), or OUKG/35/2001 (O1K-O KK and O1K-O RK), five of the six viruses with positively charged aa at both VP1 109 and 110 could be recovered using BHK-21 cells (see Table 1) and could also infect CHO cells ([32] and this study), which indicates the ability to use non-integrin receptors. However, we were surprised to find that we could not recover the O1K-Asia RK virus. This included repeated attempts to rescue the virus from transfected BHK-21 cells by passage on BTY cells. It is not clear why the O1K-Asia RK virus could not be recovered. However, the observations that the O1K-O RK and O1K-A RK viruses are viable suggest that it is unlikely that such small changes (four nucleotides and two aa) would alter vRNA translation, viral polyprotein processing, or vRNA replication, and further work will be required to identify the reasons why the O1K-Asia RK virus is non-viable. During cell culture passage, SAT viruses also accumulate aa substitutions in their capsid proteins that often involve the gain of positively charged aa at VP1 110–112. Here, we constructed chimeric viruses based on the capsids of SAT2/EGY/9/2012 with targeted aa changes at these residues (O1K-SAT2 KGR and O1K-SAT2 KKR). Both of these viruses were viable as they could be recovered using BTY cells; however, although the O1K-SAT2-KGR virus could be recovered using BTY cells, it was not recovered when using BHK-21 cells for the post-transfection passage steps. This suggests that a single R substitution at VP1 112 is tolerated in the SAT2 capsid, but it is insufficient by itself to allow the use of non-integrin receptors to initiate infection. During cell culture passage, SAT viruses also accumulate aa substitutions at VP1 83–85. A study by Maree et al. suggested that a negatively charged aa at VP1 83 could interfere with receptor interactions that involve the R at VP1 85 [26]. Therefore, based on this possibility, in the chimeric SAT2 virus generated in our study (O1K-SAT2 KHR), we introduced positively charged aa at both VP1 83 and 85. However, for reasons that we do not yet understand, this virus could not be recovered.

Importantly, the changes introduced at VP1 109–110 or 110–112 were stably maintained during cell passage for all of the recovered viruses. Furthermore, all of the recovered viruses with changes at these residues caused more rapid CPE in adherent BHK-21 cells than their corresponding parental virus, suggesting that they could spread more rapidly through the cell monolayer. However, despite causing more rapid CPE, the O1K-A KK, O1K-A RK, and O1K-Asia KK viruses only reached similar virus titres to their parental viruses (note: O1K-A WT and O1K-Asia WT could readily infect BHK-21 cells without capsid modifications). In contrast, the virus yields generated by O1K-O KK, O1K-O RK, and O1K-SAT2 KKR were dramatically increased over their parental viruses (O1K-O WT and O1K-SAT2 WT). Similar observations were made for suspension BHK-21 cells, as all of the recovered viruses with changes in VP1 (VP1 109–110 or 110–112) caused more rapid CPE than their corresponding parental virus (although this was less clear for O1K-A KK and O1K-A RK), and again, this was most dramatic for O1K-O KK, O1K-O RK, and O1K-SAT2 KKR. In line with similar virus growth in adherent BHK-21 cells, the yield of 146S intact virus particles was also similar between O1K-A KK, O1K-A RK, and O1K-Asia KK and their corresponding parental viruses (O1K-A WT and O1K-Asia WT). In contrast, and importantly for this study, in suspension BHK-21 cells, the O1K-O KK, O1K-O RK, and O1K-SAT2 KKR viruses generated significantly higher yields of intact 146S virus particles than their corresponding parental viruses. Thus, it appears that although the introduction of aa changes that allow for infection of BHK-21 cells can increase the rate of virus spread, for chimeric viruses with wt capsids (A-Turkey/2/2006 and Asia-1/Bar/9/2009) that can infect BHK-21 cells, this is not necessarily associated with concomitant increased virus yields or 146S particles. In contrast, for chimeric viruses with wt capsids (OUKG/35/2001 and SAT2/EGY/9/2012) that infect BHK-21 cells poorly, the introduction of such aa changes can greatly increase virus and 146S particle yield.

We also attempted to create a canonical HS binding site on each of the capsids selected for our study. As described above, for type O FMDV, the switch from non-HS binding to HS binding involves only a small number of aa changes in a shallow depression on the outer capsid surface. Thus, it was not surprising that the O1K-O HS virus could be recovered, as only two aa changes were required to generate the same aa sequence at the canonical HS binding site as O1BFS. The resulting virus (O1K-O HS) could infect CHO cells and caused more rapid CPE in both adherent and suspension BHK-21 cells than the parental O1K-O WT virus. However, for both cell systems, the rate of CPE was slightly slower than O1K-O KK and O1K-O RK. In addition, O1K-O HS generated greatly increased virus yields in adherent BHK-21 cells compared to O1K-O WT. Interestingly, despite causing CPE only slightly more slowly than O1K-O KK and O1K-O RK, the O1K-O HS virus gave a noticeably lower 146S yield in suspension BHK-21 cells. The identity of the receptor that is used by the O KK RK viruses to infect BHK-21 cells is yet to be identified, but it is highly likely that the HS virus uses HS receptors. Thus, it is possible that, as HS is abundantly expressed on the surfaces of most cells, the changes in the capsid may alter the propensity for the virion to remain cell associated upon cell lysis (i.e., CPE). Since the 146S yield in the suspension culture system was determined following vigorous clarification of the infected-cell lysates (to remove maximum cell debris), this could in part explain the reduced yield. It is also possible that the aa changes required to form a canonical HS binding site reduce virion stability. A study by Borca et al. [53] showed that a matched pair of viruses (based on FMDV O_1_ Campos) that differed only at VP3-56 (H or R) had similar growth kinetics in cell culture but concluded that the virus with R at VP3-56 was less temperature-stable than the virus with H at this position. However, our analysis suggests that the O1K-O HS virus (which has R at VP3-56) had a similar thermal stability to O1K-O WT (which has H at VP3-56), which suggests that different temperature sensitivities may not contribute to the lower 146S yields. A HS binding site has also been identified on the capsid of FMDV A1061. Therefore, we attempted to make a HS binding site on the chimeric virus with the capsid of A-Turkey/2/2006 by making four aa substitutions that created the same aa sequence at the HS binding site as A1061. However, for reasons that we do not yet understand, this was unsuccessful, as we could not recover a virus with these changes.

In contrast to type O and type A viruses, cell-culture-adapted Asia-1 and SAT2 viruses have not been observed with aa changes at the depression that forms the canonical HS binding site on O1BFS and A1061. This suggests that these serotypes do not have a natural propensity to generate a HS binding site at this location during cell culture adaptation. Nevertheless, we attempted to create a HS binding site on the O1K-Asia WT and O1K-SAT2 WT viruses. This involved making 7 and 10 aa changes in the capsid proteins, respectively. Given the large number of aa changes, we were not surprised to find that the O1K-SAT2 HS virus could not be rescued (including on BTY cells). In contrast, we were somewhat surprised to rescue the O1K-Asia HS virus. This virus induced rapid CPE in both adherent and suspension BHK-21 cells, which was considerably faster than for the O1K-Asia WT parental virus. Nevertheless, despite inducing faster CPE, the virus yield in adherent BHK-21 cells was not improved, and the yield of 146S particles from suspension BHK-21 cells was greatly reduced compared to O1K-Asia WT and the O1K-Asia KK variant. Thus, similarly to O1K-O HS, the yield of 146S particles was disappointing. We do not yet know the reasons for the poor yield of 146S particles but, as described above, it could be linked to an increased propensity to remain bound to abundant receptors on dead-cell membranes and/or reduced capsid stability. However, given that for O1K-Asia HS, the yield of 146S particles was extremely low, it is possible that capsid instability resulting from the introduced aa changes may provide a larger contribution to the reduced yield. Thus, our results show that increasing the rate of CPE in BHK-21 cells does not necessarily lead to an increased yield of the virus or 146S particles.

As mentioned above, full-genome sequencing indicated that all of the introduced aa changes were faithfully retained in the rescued viruses. However, for some of the rescued viruses, additional changes were observed. These mapped to the outer capsid surfaces and were located very close to the site of the introduced changes (see Figure 1). Given that the additional changes are close to the site of the introduced changes, they could directly contribute to forming a receptor-binding site that allows the infection of BHK-21 cells. With this in mind, for the O1K-Asia HS virus, the additional changes were within or close to the canonical HS binding site (VP3 H84Q and VP2 E131K), and the changes (VP3 C7R) in O1K-A KK and O1K-Asia KK viruses would further increase the number of positively charged aa at the icosahedral 5-fold symmetry axes, which could strengthen interactions with negatively charged receptors, such as heparan sulphate (or other glycosaminoglycans). Alternatively, the additional changes could indirectly influence receptor interactions or serve as capsid-stability-restoring compensatory aa substitutions [54] to offset possible reduced capsid stability that results from the introduced changes.

Importantly for vaccine efficacy, the antigenicity of most of the viruses with aa changes in the capsid was not adversely affected (Figure 3). The only exception was the O1K-SAT-KKR virus, which had a significantly lower antibody neutralisation titre when compared to the parental virus with an unmodified capsid. However, these observations were made using an antiserum that was generated using a related tissue-culture-adapted SAT2 virus, so they do not necessarily mean that a vaccine generated using the O1K-SAT2 KKR virus would not be protective against the SAT2/EGY/9/2012 field virus. Furthermore, as the aa changes introduced in our study have been seen in SAT viruses that have been adapted for growth in BHK cells [26], it is highly probable that they are present in existing vaccine viruses, which suggests that they may not adversely affect vaccine efficacy.

In summary, here we have reported a number of key findings that were obtained using chimeric viruses with capsids representative of the four most prevalent FMDV serotypes: (i) in general, chimeric viruses with capsids derived from FMDV field strains have the potential to be used in vaccine manufacture; (ii) making aa substitutions in the capsid proteins that are associated with cell culture adaptation can enhance the infection of suspension BHK cells, which are used for virus propagation for vaccine manufacture; (iii) for viruses with wt capsids (derived from field strains) that can infect BHK cells without the need for modification, the introduction of aa substitutions associated with cell culture adaptation can increase the rate of CPE in BHK suspension cells, but this is not always accompanied by an increased yield of 146S particles; and (iv) for viruses with wt capsids (derived from field strains) that poorly infect BHK cells, the introduction of aa substitutions associated with cell culture adaptation can dramatically increase the rate of CPE and yield of 146S particles in BHK suspension cells. In addition, our results show that viruses with aa substitutions that map to the icosahedral, 5-fold symmetry axes of the capsid give greater yields of 146S particles than viruses with aa substitutions at the depression that forms the canonical HS binding site. Surprisingly, this appears to also include type O FMDV, which preferentially acquires aa substitutions at the canonical HS binding site during cell culture adaptation.

## 5. Conclusions

These findings could be used to reduce the time taken for the cell culture adaptation of FMDV field strains. In addition, generating vaccine viruses by means of reverse genetics could also reduce the need for extraneous agent testing when vaccine viruses are produced directly from field isolates. These techniques could prove invaluable in speeding up the production of new vaccine strains that are urgently needed to provide protection against emerging field viruses for which existing vaccines provide a poor match.

## Figures and Tables

**Table 1:** Sequences of the chimeric viruses (type A, O and Asia-1) with amino acid changes at the 5-fold symmetry axes (VP1 109–110). The virus names indicate viruses with wild type (WT) or modified capsids. The field strains from which the capsids are derived are indicated. The amino acid changes are indicated in red. The ability to recover each virus from the appropriate infectious copy plasmid by post transfection passage on BHK or BTY cells is shown with a ✓ or ✘. ND = Not done.

Name	Capsid (VP2-2A)	VP1 109-110	Recovery	
			BHK	BTY
O1K-A WT	A-Turkey/2/2006	KQ	ND	✓
O1K-A KK	A-Turkey/2/2006	K**K**	✓	ND
O1K-A RK	A-Turkey/2/2006	**RK**	✓	ND
O1K-Asia WT	Asia-1/Bar/9/2009	KQ	ND	✓
O1K-Asia KK	Asia-1/Bar/9/2009	K**K**	✓	ND
O1K-Asia RK	Asia-1/Bar/9/2009	**RK**	✘	✘
O1K-O WT	OUKG/35/2001	KA	ND	✓
O1K-O KK	OUKG/35/2001	K**K**	✓	✘
O1K-O RK	OUKG/35/2001	**RK**	✓	✘

**Table 2:** Sequences of the chimeric SAT2 viruses with amino acid changes at the 5-fold symmetry axes (VP1 83–85 and 110–112). The virus names indicate wild type (WT) or modified capsids. The field strain from which the capsid was derived is indicated. The amino acid changes are indicated in red. The ability to recover each virus from the appropriate infectious copy plasmid by post transfection passage on BHK or BTY cells is shown with a ✓ or ✘. ND = Not done.

Name	Capsid (VP2-2A)	VP1 110-112	VP1 83-5	Recovery	
				BHK	BTY
O1K-SAT2 WT	SAT2/EGY/9/2012	KGG	DHT	ND	✓
O1K-SAT2 KGR	SAT2/EGY/9/2012	KG**R**	DHT	✘	✓
O1K-SAT2 KKR	SAT2/EGY/9/2012	K**KR**	DHT	✓	ND
O1K-SAT2 KHR	SAT2/EGY/9/2012	KGG	**K**H**R**	✘	✘

**Table 3:** Sequences of the chimeric viruses with amino acid changes at the canonical HS binding site. The sequence is shown for the cell-culture-adapted strains O1BFS and A1061 and for the chimeric viruses with field strain capsids (WT) or with changes introduced at residues that line the canonical HS binding site. The amino acid changes are indicated in red. Note: The C terminal residues are structurally equivalent but numbered differently due to different lengths of VP1. Similarly, the second motif in VP3 located at residues 84–88 in A1061 and O1BFS is located at residues 83–87, marked with an asterisk (*) due to the different lengths of VP3. The ability to recover each virus from the appropriate infectious copy plasmid by post transfection passage on BHK or BTY cells is shown with a ✓ or ✘. ND = Not done.

**Name**	**Capsid (VP2-2A)**	**VP2 134-138**	**VP3** **55-60**	**VP3** **84-88 or** 83-87 *	**VP1-C-Term**	**Recovery**	
						**BHK**	**BTY**
**A1061**	**A1061**	**TREKY**	**LRFDDG**	**KHMSN**	**KVT (193–195)**		
O1K-A WT	A-Turkey/2/2006	PREKY	LCFDDG	KHMSN	EVL (194–196)	ND	✓
O1K-A HS	A-Turkey/2/2006	**T**REKY	L**R**FDDG	KHMSN	KV**T** (194–196)	✘	✘
O1K-Asia WT	Asia-1/Bar/9/2009	TRQKY	LRFGEV	GHMSN *	DTT (193–195)	ND	✓
O1K-Asia HS	Asia-1/Bar/9/2009	TR**E**KY	LRF**DDG**	**K**HMSN *	**KV**T (193–195)	✓	ND
O1K-SAT2 WT	SAT2/EGY/9/2012	AREEF	LNFDGK	KVGHN *	KHT (198–200)	ND	✓
O1K-SAT2 HS	SAT2/EGY/9/2012	**T**RE**KY**	L**R**FD**DG**	K**HMS**N *	K**V**T (198–200)	✘	✘
**01BFS**	**01BFS**	**KRELY**	**LRFEGG**	**KHMSN**	**HPT (195** **–197)**		
O1K-O WT	OUKG/35/2001	KRELY	LHFEGG	KHMSN	HPS (195–197)	ND	✓
O1K-O HS	OUKG/35/2001	KRELY	L**R**FEGG	KHMSN	HP**T** (195–197)	✓	ND

**Table 4:** Virus titres for the recovered viruses on BTY and BHK cells**.** Virus titres (pfu/mL) at BHK p4 (modified viruses) or BTY p4 (WT viruses) were determined by plaque assay. For each virus, titres are shown for BTY and BHK cells. The right-hand column shows the BTY/BHK titre ratio. For O1K-SAT2 WT on BHK, no plaques were seen at 1/10 dilution using an inoculum of 100 μL; therefore, only the BTY/BHK ratio for this virus could be accurately determined.

Virus	BHK Titre (PFU/mL)	BTY Titre (PFU/mL)	Ratio BTY/BHK Titre
O1K-A WT	2.0 × 10^6^	3.6 × 10^7^	18.0
O1K-A KK	5.5 × 10^6^	8.5 × 10^6^	1.5
O1K-A RK	1.8 × 10^7^	5.0 × 10^7^	2.8
			
O1K-Asia WT	2.0 × 10^6^	2.3 × 10^7^	11.5
O1K-Asia HS	7.5 × 10^6^	2.3 × 10^7^	3.1
O1K-Asia KK	1.3 × 10^7^	4.8 × 10^7^	3.7
			
O1K-O WT	7.5 × 10^3^	1.6 × 10^7^	2133.3
O1K-O HS	8.9 × 10^7^	1.1 × 10^8^	1.2
O1K-O KK	2.4 × 10^7^	5.0 × 10^7^	2.1
O1K-O RK	1.9 × 10^7^	5.0 × 10^7^	2.6
			
O1K-SAT2 WT	<1.0 × 10^2^	2.0 × 10^6^	>20,000
O1K-SAT2 KKR	5.5 × 10^6^	4.6 × 10^7^	8.4

## Data Availability

The datasets used and/or analysed during the current study are available from the corresponding author on reasonable request.
